# SIRT1 reduction causes renal and retinal injury in diabetes through endothelin 1 and transforming growth factor β1

**DOI:** 10.1111/jcmm.12557

**Published:** 2015-03-06

**Authors:** Rokhsana Mortuza, Biao Feng, Subrata Chakrabarti

**Affiliations:** Department of Pathology, Schulich School of Medicine & Dentistry, University of Western OntarioLondon, ON, Canada

**Keywords:** endothelial cells, SIRT1, ET-1, TGF-β1, p300

## Abstract

In diabetes, hyperglycaemia causes up-regulation of endothelin 1 (ET-1) and transforming growth factor beta 1 (TGF-β1). Previously we showed glucose reduces sirtuin1 (SIRT1), a class III histone deacetylase. Here, we investigated the regulatory role of SIRT1 on ET-1 and TGF-β1 expression. Human microvascular endothelial cells were examined following incubation with 25 mmol/l glucose (HG) and 5 mmol/l glucose (NG) with or without SIRT1 or histone acetylase p300 overexpression or knockdown. mRNA expressions of ET-1, TGF-β1, SIRT1, p300 and collagen 1α(I) were examined. SIRT1 enzyme activity, ET-1 and TGF-β1 protein levels were measured. Histone acetylation and endothelial permeability were further investigated. Similar analyses were performed in the kidneys and retinas of SIRT1 overexpressing transgenic mice with or without streptozotocin induced diabetes. Renal functions were evaluated. In the endothelial cells (ECs), HG caused increased permeability and escalated production of ET-1, TGF-β1, collagen Iα(I). These cells also showed increased p300 expression, histone acetylation and reduced SIRT1 levels. These changes were rectified in the ECs following p300 silencing or by SIRT1 overexpression, whereas SIRT1 knockdown or p300 overexpression in NG mimicked the effects of HG. High ET-1 and TGF-β1 levels were seen in the kidneys and retinas of diabetic mice along with micro-albuminuria and increased fibronectin protein (marker of glucose-induced cell injury) levels. Interestingly, these detrimental changes were blunted in SIRT1 overexpressing transgenic mice with diabetes. This study showed a novel SIRT1 mediated protection against renal and retinal injury in diabetes, regulated through p300, ET-1 and TGF-β1.

## Introduction

Diabetes and its long-term chronic complications are a growing concern worldwide 1 as the number of people being diagnosed with diabetes in North America is increasing in an alarming rate 1. In diabetes, dysfunction of the vascular endothelium caused by hyperglycaemia is a key initiating factor in the development of all chronic diabetic complications including diabetic nephropathy and retinopathy 2,3. A structural hallmark of such chronic diabetic complication is vascular basement membrane thickening and renal mesangial expansion. This is caused by an increased production of extracellular matrix (ECM) proteins such as fibronectin (FN) and collagen. Although other cells may take part in such process, endothelial cells (ECs) lining the blood vessels play an important role 2–5.

In the ECs, glucose-induced increased oxidative stress activates several signalling pathways altering crucial gene expressions of several growth, vasoactive and transcription factors 6,7. Endothelin 1 (ET-1) and transforming growth factor β1 (TGF-β1) are two such vasoactive and growth factors, which have been implicated in the development and progression of several vascular diseases including diabetes 8–14. We and others have previously shown that ET-1 and TGF-β1 are increased in ECs, organs of diabetic animals and in circulation of patients with diabetes 2,8–14.

Endothelin 1 is a peptide produced and secreted by the vascular ECs and is crucial for maintaining vascular tone, integrity and homeostasis 8–13. Endothelin 1 is a potent mitogen which influence local cellular growth and survival. Because of its potent mitogenic and vasoconstrictor functions, ET-1 can play a major pathogenic role in diabetic complications. Hyperglycaemia induced increased ET-1 causes vascular damage by altering blood flow, increasing vascular permeability and by increasing ECM proteins (such as FN, collagen) productions as shown by us and others previously 8–13. Transforming growth factor β1 is a secreted cytokine produced by many cells including ECs, belonging to a large family of regulatory proteins that control cell growth, proliferation, differentiation and apoptosis 8–14. In recent years, TGF-β1 has been evolved as one vital regulator of ECM protein production in diabetes 2,8–14. Increased TGF-β1 alters ECM protein synthesis and degradation, a delicate balance that is lost in diabetes causing fibrosis in the affected tissues 2,14. We have previously shown that diabetes-induced up-regulated ET-1 and TGF-β1 levels causes increased production of ECM proteins in ECs and tissues of diabetic animals 2,8–14. Moreover, we have demonstrated transcriptional co-activator and histone acetyl transferase (HAT) p300 interacts with transcription factors NFκB and AP-1 to regulate the expression of these genes 15–19.

SIRTs (silent information regulator proteins) are a group of NAD dependent class III histone deacetylases that regulate epigenetic gene silencing. SIRTs deacetylase not only histones but also many proteins and transcription factors. SIRT1 is a prominent enzyme in the SIRT family residing both in the cytoplasm and nucleus. SIRT1 has been found to play crucial role in cellular oxidative stress response, metabolism, differentiation, longevity and various diseases including diabetes 20–23. We have previously shown that glucose induced increased oxidative stress causes reduction of SIRT1 in ECs and tissues of animals with diabetes causing rapid ageing, reduced antioxidants and increased FN production in diabetes 20. Moreover SIRT1 being a deacetylase also has a regulatory relationship with p300 20.

We investigated the role of SIRT1 and its relationship with p300 in regulating ET-1 and TGF-β1 and their possible downstream consequences in ECs and tissues of animals affected by diabetes. To this extent, we used human microvascular ECs (HMECs) for *in vitro* inspections of glucose-induced endothelial alterations. We further expanded the study using SIRT1 overexpressing transgenic mice in which we induced diabetes with streptozotocin (STZ) and examined their renal and retinal tissues.

## Materials and methods

### Cell culture

Dermal-derived HMEC was obtained (Lonza, Walkersville, MD, USA) and grown in endothelial cell basal medium 2 (EBM-2; Lonza). Human embryonic kidney (HEK293A) cells were obtained from ATCC (Manassas, VA, USA). We have previously described the culture conditions of these cells 24–27. The glucose concentration in the growth medium was 5 mmol/l. No insulin was present in any media. All cells were maintained in a humidified atmosphere containing 5% CO_2_ and at 37°C incubation. Cells were treated with normal glucose (NG, 5 mmol/l) or high glucose (HG, 25 mmol/l d-glucose) or osmotic control (OSM, 25 mmol/l l-glucose). All reagents were obtained from Sigma Chemicals (Sigma-Aldrich, Oakville, ON, Canada) unless specified otherwise. Experiments were conducted with 6–10 biological replicates.

### Animal experiments

Wild-type (C57BL/6J) and SIRT1 transgenic mice (C57BL/6-Actb^tm3.1(Sirt1)Npa^/J), male, 8 weeks old were obtained (Jackson Laboratory, Bar Harbor, ME, USA) and genotyped with Real Time RT-PCR. Diabetes was induced by single intra-peritoneal injection of STZ (65 mg/kg in citrate buffer). Streptozotocin (Sigma-Aldrich, Burlington, ON, Canada) was prepared in cold 0.1 M citrate buffer (pH 4.5) fresh before injection. Age and sex matched mice were used as controls and received equal volume of citrate buffer. Diabetes was defined in the animals with blood glucose level >20 mmol/l on two consecutive days with Freestyle Freedom Lite blood glucose monitoring system (Abbott Diabetes Care, Saint-Laurent, QC, Canada). The animals were fed on a standard rodent diet with water *ad libitum* and killed after 2 months following the development of diabetes (*n* = 10/group). Urinary micro-albumin (Exocell, Philadelphia, PA, USA) was measured as per the kit instructions. Retina and kidney tissues were collected and snap frozen in liquid nitrogen for histology. All tissues were stored at −80°C until further analysis.

All animal experiments were performed in accordance with regulations specified by the Canadian Council of Animal Care. All protocols were approved by the University of Western Ontario Animal Care and Veterinary Service. The investigation was in compliance with the Guide for the Care and Use of Laboratory Animals (NIH publ. no. 85-23, revised 1996).

### SIRT1 enzyme activity assay

Whole cell lysate were collected following treatment with RIPA buffer (Millipore, MA) with addition of protease inhibitor (Roche, Laval, QC, Canada). The total protein concentrations of the samples measured using a commercially available BCA protein assay kit (Pierce, Rockford, IL, USA). Enzyme assay for SIRT1 activity was performed as per the manufacturer instructions (Sigma-Aldrich). The plates were read with a fluorescent spectrophotometer (Biotek, Winooski, VT, USA) at excitation 340 nm and emission 430 nm.

### mRNA extraction and cDNA synthesis

RNA from cells was isolated using TRIZOL™ (Invitrogen, Burlington, ON, Canada) reagent as established at our laboratory. Briefly RNA was extracted with chloroform followed by centrifugation to separate the sample into aqueous and organic phases. The RNA was recovered from the aqueous phase by isopropyl alcohol precipitation and suspended in DEPC water. RNA concentration was assessed on a spectrophotometer (Gene Quant-Pharmacia Biotech, Cambridge, MA, USA). First-strand cDNA was made by using High Capacity cDNA Reverse Transcription kit (Applied Biosystems, Foster City, CA, USA) according to the manufacturer instruction. The resulting products were stored at −20°C until further analysis by real time RT-PCR.

### mRNA analysis with real time RT-PCR

Real time RT-PCR was performed in LightCycler™ (Roche Diagnostics) to quantify the mRNA expression of SIRT1 and using the Qiagen One Step RTPCR kit (detection platform SYBR Green I). All of the primers were either ordered or custom made from Sigma-Aldrich (Table1). For a final reaction volume of 20 μl, the following reagents were added: 4.4 μl of H_2_O, 10 μl of SYBR (Sigma-Aldrich), 1.6 μl of MgCl_2_, 1 μl forward/reverse primer and 2 μl of cDNA. To optimize the amplification of the genes, melting curve analysis was used to determine the melting temperature (*T*_m_) of specific products and primer dimers. According to the *T*_m_ value of specific products for respective genes, an additional step (signal acquisition step, 2–3°C below *T*_m_) was added following the elongation phase of RT-PCR. The data were analysed using the standard curve method (*R*^2^ > 0.98, amplification efficiency>90%). The data were normalized to housekeeping gene 18sRNA to account for differences in reverse transcription efficiencies and the amount of template in the reaction mixtures.

**Table 1:** Oligonucleotide sequences for real-time PCR

Name of primer	Sequence (5′–3′)
Collagen Iα(I) (human)	GAGGGCCAAGACGAAGACATC
	CAGATCACGTCATCGCACAAC
ET-1 (human)	AAGCCCTCCAGAGAGCGTTAT
	CGAAGGTCTGTCACCAATGT
	6FAM-TGACCCACAACCGAG-GBNFQ
ET-1 (mice)	TTAGCAAGACCATCTGTGTG
	GAGTTTCTCCCTGAAATGTG
SIRT1 (human/mice)	GCAGATTAGTAGGCGGCTTG
	TCTGGCATGTCCCACTATCA
p300 (human)	GGGACTAACCAATGGTGGTG
	ATTGGGAGAAGTCAAGCCTG
p300 (mice)	AGGCAGAGTAGGACAGTGAA
	CTCAGTCTGGGTCACTCAAT
TGF-β1 (human)	GCCCACTGCTCCTGTGACA
	CGGTAGTGAACCCGTTGATGT
	6FAM-CAGGGATAACACACTGC-MGBNFQ
TGF-β1 (mice)	TGGAGCAACATGTGGAACTC
	GTCAGCAGCCGGTTACCA
18s (human/mice)	GTAACCCGTTGAACCCCATT
	CCATCCAACGGTAGTAGCG

### Adenoviral overexpression of SIRT1 and SIRT1 gene silencing

SIRT1 adenovirus was obtained (ABM, Richmond, BC, Canada) and amplified in HEK293A cells. HMECs were transfected with the adenovirus as described earlier 24. To silence SIRT1, transfection of small interfering RNA (siRNA) was performed using N-TER nanoparticle siRNA transfection system (Sigma-Aldrich) as described previously 20,27. To silence SIRT1 a combination of two different siRNAs from Dharmacon Inc. (Lafayette, CO, USA) and Santa Cruz Biotechnology (Santa Cruz, CA, USA) was used to minimize the off target effects 20,27. Transfection efficiency was assessed by real time RT-PCR.

### p300 gene silencing and p300 overexpression

A combination of four different siRNAs prepared in house was used to specifically silence the p300 expression in ECs as described by us previously 19,20. p300 overexpression was achieved with expression vectors (generously provided by Dr. Joan Boyes, The Institute of Cancer Research, London, UK) containing the human wild-type p300 (pCI-p300) and its HAT-deletion mutant (pCI-p300 HATΔ1472–1522) as described earlier 15. Transfection efficiency was assessed by measuring p300 mRNA expression by real time RT-PCR.

### ET-1, TGF-β1 & FN ELISA

Human/mice ET-1 ELISAs (Enzo, Farmingdale, NY, USA) and TGF-β1 ELISAs (eBioscience, San Diego, CA, USA) were done on cell and tissue lysates as per the manufacturer instructions. FN ELISAs (Abcam, Toronto, ON, Canada) were done on mice tissue lysates as per the manufacturer instructions. The total protein concentrations were measured by BCA protein assay kit (Pierce). The plate was read at 450 nm using a plate reader (Multiskan, Thermofisher, Toronto, ON, Canada).

### Total ROS/RNS assay

Total ROS/RNS (reactive oxygen species/reactive nitrogen species) assay (Cell Biolab, San Diego, CA, USA) on tissue lysate was measured as per the manufacturer instructions. The plates were read with a fluorescent plate reader (Biotek) at excitation 480 nm and emission 530 nm.

### Endothelial permeability assay

Human microvascular endothelial cells were seeded onto inserts (1 μm pores) in 24-well plates, with or without incubation with specific reagents for 24 hrs, and were tested for vascular permeability using the In Vitro Vascular Permeability Assay Kit (Millipore, Billerica, MA, USA) according to the manufacturer’s instructions. Endothelial cells were seeded at a concentration of 2 × 10^6^ cells/ml (200 μl/insert). Cell morphology and confluency was monitored under the microscope and upon formation of monolayer following 72 hrs, cells were treated with specific reagents (Ad-SIRT1, p300 siRNA, p300 plasmid, SIRT1 siRNA) with or without glucose. Following treatment FITC-Dextran is added to the cells and permeability of this fluorescent molecule is measured at several intervals (0.5, 1, and 2 hrs) by measuring the fluorescence of the receiver plate well solution. The plates were read with a fluorescent spectrophotometer (Biotek) at excitation 485 nm and emission 535 nm.

### Western blotting

The western blot analysis was conducted according to the standard protocol established at our lab 15 using acetylated H3 lysine (Epigentek, Farmingdale, NY, USA) & β-actin antibody (Santa Cruz Biotechnology).

### Statistical analysis

Data are expressed as mean ± SEM, normalized to controls. The statistical significance of the results was analysed by one-way or two-way anova followed by Tukey’s HSD post hoc correction and the two tailed Students *t*-test as appropriate (PASW Statistics 18; IBM, Markham, ON, Canada). A *P*-value <0.05 was considered statistically significant.

## Results

### SIRT1 regulates ET-1 and TGF-β1 expressions in the ECs in high glucose

**Figure 1:**
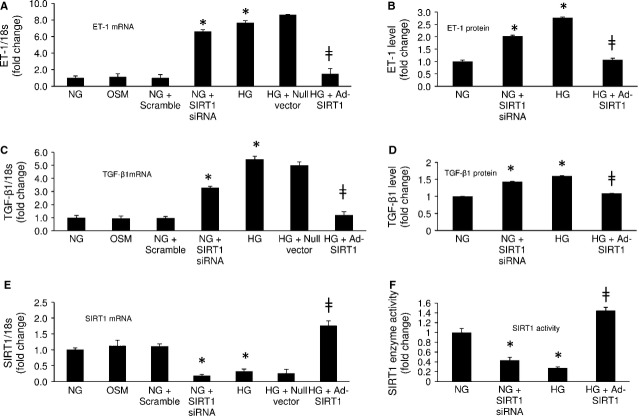
SIRT1 regulates ET-1 and TGF-β1 expression in ECs. Human microvascular endothelial cells (HMECs) exposed to 25 mmol/l (HG) glucose compared to 5 mmol/l glucose (NG) showed increased (A) ET-1 mRNA, (C) TGF-β1 mRNA and reduced (E) SIRT1 mRNA expressions. Such changes were not seen when the cells were incubated with 25 mmol L-glucose (osmotic control, OSM). Transfection of endothelial cells with Ad-SIRT1 (but not the null vector) increased (F) the enzyme activity in treated cells and normalized glucose-induced up-regulation of (A and C) ET-1 and TGF-β1 mRNA and (B and D) protein levels. Glucose-like effects (ET-1, TGF-β1up-regulation) were further seen when cells in NG were transfected with SIRT1 siRNA (A, C, E) but not with scramble siRNA. Such knockdown of SIRT1 significantly reduced (F) the enzyme activity in NG and increased the (B) ET-1 and (D) TGF-β1 protein levels in these cells. * = significantly different from NG, ╪ = significantly different from HG, mRNA levels are expressed as a ratio of 18s. All data (mean ± SEM, *P* < 0.05) were normalized to NG; *n* = 4–6/group.

As microvascular ECs are the major target of glucose-induced organ damage, we investigated HMECs exposed to 5 (NG) and 25 (HG) mmol/l glucose for 72 hrs. These concentrations are based on our previous dose dependent analysis of SIRT1 levels 20,24. Quantitative real time RT-PCR analysis of the ECs showed significant up-regulation of ET-1 and TGF-β1 mRNA levels (Fig.1A and C) and down-regulation of SIRT1 mRNA levels (Fig.1E) with HG compared to NG. No effects were seen following incubation with osmotic control (25 mmol/l of L-glucose, OSM; Fig.1A, C and E). These findings were further reflected in ET-1 and TGF-β1 protein analysis and SIRT1 enzyme activity analysis, which showed a marked increase of ET-1 and TGF-β1 protein levels (Fig.1B and D) and reduced SIRT1 enzyme activity levels (Fig.1F) in these cells following HG treatment (compared to NG).

We then proceeded to explore any relationship, between the observed glucose-induced down-regulation of SIRT1 and up-regulation of ET-1 and TGF-β1 expressions in these ECs. For this purpose, we analysed the ECs following transfection with SIRT1 adenovirus (Ad-SIRT1). Transfection efficiency was measured with real time RT-PCR and confirmed with SIRT1 enzyme analysis (Fig.1E and F). We noted the Ad-SIRT1 transfection significantly increased the SIRT1 mRNA and enzyme levels in the ECs in HG (Fig.1E and F). Most importantly, this transfection caused a significant reduction in ET-1 and TGF-β1 mRNA and protein levels in these ECs in HG (Fig.1A–D). Endothelial cells transfected with null vector did not show such change (Fig.1A and C).

In addition, SIRT1 knockdown with siRNA (efficiency in Fig.1E and F) in NG mimicked the effects of HG, causing an increase in ET-1 and TGF-β1 levels in these ECs (Fig.1A–D). Conversely, ECs transduced with scramble siRNA did not show these changes (Fig.1A–D). These results demonstrated a SIRT1 mediated regulation of ET-1 and TGF-β1 in the ECs in hyperglycaemia.

### SIRT1 regulates ET-1 and TGF-β1 expressions *via* p300

**Figure 2:**
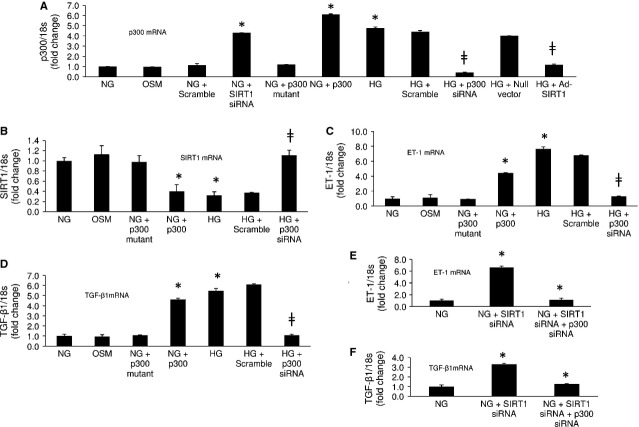
SIRT1 regulates ET-1 and TGF-β1 *via* p300. HG caused increased (A) p300 mRNA and reduced (B) SIRT1 mRNA levels in ECs. (A) Ad-SIRT1 transfection in HG prevented p300 up-regulation and SIRT1 knockdown with siRNA in NG up-regulated p300 mRNA levels. (B) p300 siRNA transfection in HG (efficiency in A) normalized SIRT1 expression. Furthermore, p300 overexpression in NG (efficiency in A) reduced SIRT1 mRNA levels. (C and D) HG induced ET-1 and TGF-β1 up-regulation was abrogated with p300 siRNA (not with scramble) transfection. p300 overexpression in NG up-regulated the expressions of these transcripts. (E and F) Rescue experiment showing up-regulation of ET-1 and TGF-β1 mRNA levels in NG with SIRT1 knockdown can be resolved with subsequent p300 knockdown in the ECs. NG = 5 mmol/l glucose, HG = 25 mmol/l glucose, OSM = osmotic control, * = significantly different from NG, ╪ = significantly different from HG, mRNA levels are expressed as a ratio of 18s. All data (mean ± SEM, *P* < 0.05) were normalized to NG; *n* = 4–6/group.

In order to investigate the mechanism of such SIRT1 mediated ET-1 and TGF-β1 regulation we looked at transcriptional co-activator p300, as we previously have found SIRT1 and p300 balance each other 20. In keeping with the earlier findings we noted SIRT1 overexpression with adenovirus in 25 mmol/l glucose normalizes glucose-induced augmented p300 mRNA levels (Fig.2A). On the contrary, SIRT1 knockdown in NG with siRNA caused an increase in p300 mRNA levels (Fig.2A). Furthermore, p300 overexpression in these cells in NG reduced the SIRT1 mRNA levels (Fig.2B and C). These changes were not seen with null vector, scramble siRNA and p300 mutant transfections. These results re-established a regulatory and balancing role of SIRT1 and p300 on each other in ECs.

To confirm the downstream effects of such changes we examined the cells following knockdown of p300 with siRNA (efficiency in Fig.2B) in HG. Such treatment not only normalized the SIRT1 mRNA levels (Fig.2C) but also reduced the ET-1 and TGF-β1 mRNA levels in HG (Fig.2D and E) compared to scrambled controls. In addition, p300 overexpression in NG lead to an increased mRNA levels for both ET-1 and TGF-β1 mimicking the HG treatments (Fig.2D and E). p300 mutant transfected cells did not show such change. ET-1 and TGF-β1 protein levels paralleled the mRNA findings (Fig. S1A and B). These results further establish that SIRT1 regulates ET-1 and TGF-β1 through p300.

We further conducted several rescue experiments to confirm such pathway. We first knocked down SIRT1 with siRNA in NG in these ECs, following which we transfected the cells with p300 siRNA. Transfection efficiency assessed with real time RT-PCR showed reduction of both SIRT1 (88%) and p300 (61%) mRNA levels in the treated cells. We noted such transfection prevented the ET-1 and TGF-β1 mRNA up-regulation in the treated cells (Fig.2F). Moreover p300 overexpression in HG following treatment with Ad-SIRT1 reversed the beneficiary effects of SIRT1 causing up-regulation of ET-1 and TGF-β1 mRNA levels (Fig. S1C and D). On the other hand p300 knockdown in these cases did not alter the beneficiary effects of SIRT1 normalizing ET-1 and TGF-β1 mRNA levels (Fig. S1C and D).

### SIRT1 overexpression prevents glucose-induced increased endothelial permeability and collagen Iα(I) expression

**Figure 3:**
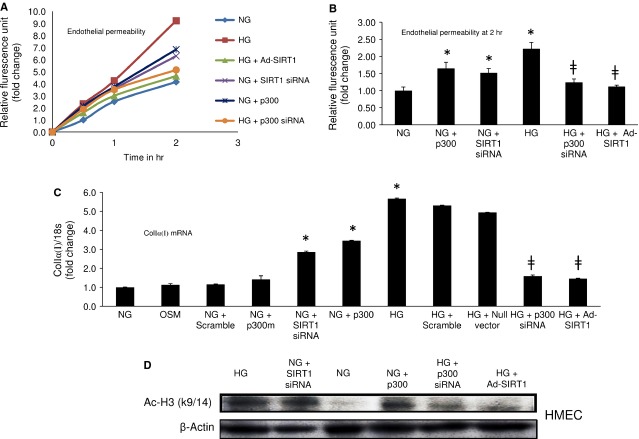
SIRT1 overexpression prevents glucose-induced increased endothelial permeability and collagen Iα(I) expression. (A) Duration dependent endothelial permeability and (B) end-point analysis showed, HG induced increased endothelial permeability was prevented by Ad-SIRT1 transfection or p300 siRNA treatment. SIRT1 siRNA or p300 overexpression in NG caused increased permeability by these cells mimicking the effects of HG. (C) HG caused an increase in Collagen Iα(I) mRNA expression in the ECs which was prevented by Ad-SIRT1 or p300 siRNA transfection. Opposingly, SIRT1 knockdown or p300 overexpression both lead to an up-regulation of Collagen Iα(I) mRNA levels in NG. (D) Western blot analysis of acetylated histone (Ac-H3K9/14) shows Ad-SIRT1 or p300 siRNA transfection reduced HG-induced increased histone acetylation in HMECs. On the other hand SIRT1 siRNA or p300 overexpression increased such acetylation in NG in these ECs. NG = 5 mmol/l glucose, HG = 25 mmol/l glucose, * = significantly different from NG, ╪ = significantly different from HG. HMECs  =  human microvascular endothelial cells. mRNA levels are expressed as a ratio of 18s. All data (mean ± SEM, *P* < 0.05) were normalized to NG; *n* = 4–6/group.

To investigate the functional consequences of SIRT1 mediated ET-1 and TGF-β1 regulation we conducted trans-endothelial permeability assay as increased endothelial permeability is a characteristic alteration in early diabetic microangiopathy. Data from the *in vitro* permeability test showed that HG significantly increases ECs permeability and Ad-SIRT1 transfection prevented such leakage in these cells (Fig.3A and B). p300 siRNA transfection in HG also had similar effect showing reduction in glucose-induced leakage whereas; SIRT1 knockdown or p300 overexpression in NG both significantly increased the ECs permeability mimicking the HG (Fig.3A and B). In addition, p300 overexpression in HG following treatment with Ad-SIRT1 reversed the beneficiary effects of SIRT1 causing increased EC permeability whereas p300 knockdown in the same scenario did not alter the beneficiary effects of SIRT1 (Fig. S1E).

Previously we have shown SIRT1 overexpression prevented glucose-induced FN up-regulation in the ECs 27. We further examined collagen Iα(I) expression levels following HG exposure. With Ad-SIRT1 transfection, we observed a normalization of glucose-induced up-regulation of collagen Iα(I) mRNA levels (Fig.3C). HG induced increased collagen Iα(I) mRNA expression was further prevented by p300 siRNA transfection (Fig.3C). In parallel, SIRT1 knockdown or p300 forced expression both caused an up-regulation of collagen Iα(I) mRNA levels in NG (Fig.3C). Moreover, we conducted western blot analysis of acetylated histone (Ac-H3K9/14) to see the functional consequences of p300 and SIRT1 alteration. Such analysis showed that HG-induced increased Ac-H3K9/14 was reduced with Ad-SIRT1 or p300 siRNA transfection (Fig.3D). Opposingly, SIRT1 siRNA or p300 forced expression in NG increased such acetylation in the ECs. These experiments together provide further evidence that SIRT1 regulate ET-1 and TGF-β1 through p300.

### SIRT1 regulates ET-1 and TGF-β1 expressions in the kidney and retina of diabetic mice

**Figure 4:**
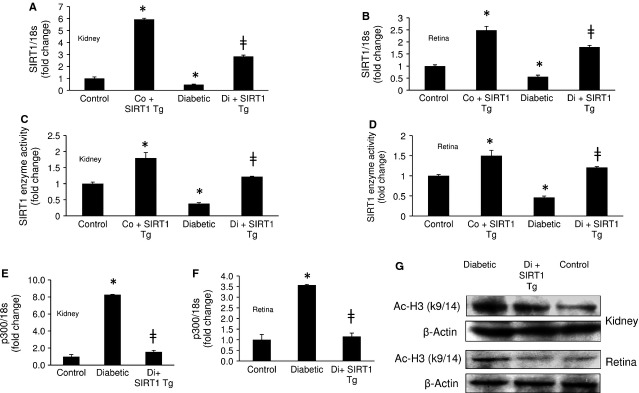
SIRT1 overexpressing diabetic mice shows reduced p300 expressions in the kidney and retina. Transgenic mice with SIRT1 overexpression (SIRT1 Tg) showed increased expressions of (A and B) SIRT1 mRNA levels and (C and D) enzyme activity in the kidneys and retinas of diabetic and control animals. Such overexpression averted diabetes-induced increased (E and F) p300 mRNA levels and (G) histone acetylation (Ac-H3K9/14) in these tissues. Control = Non-diabetic wild-type, Co+SIRT1 Tg = SIRT1 transgenic mice non-diabetic, Diabetic = Wild-type diabetic, Di+SIRT1 Tg = SIRT1 transgenic mice diabetic, Co = Control, Di =Diabetic, * = significantly different from control, ╪ = significantly different from Diabetic. mRNA levels are expressed as a ratio of 18s. All data (mean ± SEM, *P* < 0.05) were normalized to Co; *n* = 8–10/group).

Following the establishment that SIRT1 regulated ET-1 and TGF-β1 in ECs, we expanded our study to investigate whether the mechanisms seen in these cells was important in the development of renal and retinal microangiopathy in a well-established animal model. Streptozotocin-induced diabetic mice showed hyperglycaemia (diabetics 21.91 ± 4.58 mmol/l *versus* controls 7.38 ± 0.91 mmol/l, *P* < 0.001) and reduced bodyweight (diabetics 22.80 ± 1.40 g *versus* controls 30.25 ± 2.36 g, *P* < 0.001). Initially we performed real time RT-PCR analysis of the renal and retinal tissue from these mice following 2 months of uncontrolled diabetes. We have previously shown that diabetes induced increased ECM protein and vasoactive factor expression is established at this time-point 19,20,24–27. mRNA analysis showed that diabetic mice had significant down-regulation of SIRT1 expressions in the kidneys and retinas (Fig.4A and B). This finding was further reflected in the SIRT1 enzyme activity levels (Fig.4C and D). Moreover, these mice showed significant up-regulation of p300 mRNA levels in the renal and retinal tissues which were abrogated in SIRT1 overexpressing diabetic mice (Fig.4E and F). Western blot analysis further showed that SIRT1 overexpression caused reduction in histone acetylation (Ac-H3K9/14) in these tissues demonstrating the downstream functional consequences of p300 alteration in these tissues (Fig.4G). Absence of Rd8 mutation 28 in the transgenic mice were confirmed with genotyping (Fig. S2A). Microscopic examination of renal and retinal tissues showed no morphologically evident lesions (Fig. S2B).

**Figure 5:**
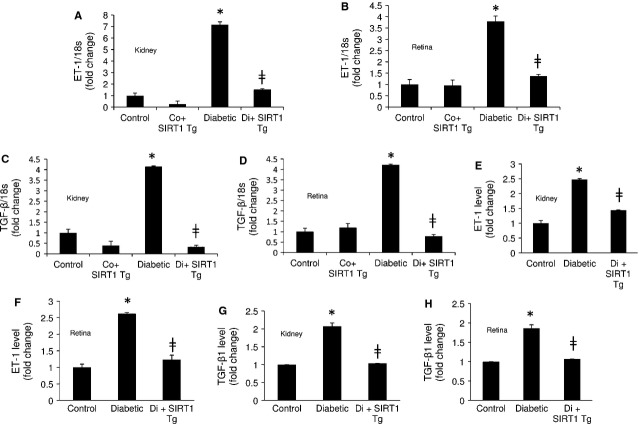
SIRT1 regulates ET-1 and TGF-β1 expressions in kidney and retina of diabetic mice. SIRT1 overexpression was protective against diabetes-induced (A and B) ET-1 and (B and C) TGF-β1 mRNA up-regulation in the kidneys and retinas of mice. In keeping with the mRNA expressions SIRT1 transgenic mice (SIRT1 Tg) with diabetes showed reduced (E and F) ET-1 and (G and H) TGF-β1 protein levels in these tissues compared to the wild-type diabetic mice. Control = Non-diabetic wild-type, Co+SIRT1 Tg = SIRT1 transgenic mice non-diabetic, Diabetic = Wild-type diabetic, Di+SIRT1 Tg = SIRT1 transgenic mice diabetic, Co = Control, Di = Diabetic, * = significantly different from control, ╪ = significantly different from Diabetic. mRNA levels are expressed as a ratio of 18s. All data (mean ± SEM, *P* < 0.05) were normalized to Co; *n* = 8–10/group.

We further looked at ET-1 and TGF-β1 levels in these mice. SIRT1 overexpressing diabetic mice showed significantly reduced level of ET-1 and TGF-β1 mRNA levels compared to diabetic mice in both tissues (Fig.5A–D). We further confirmed these results using ELISA which showed diabetes-induced increased ET-1 and TGF-β1 protein levels in the renal and retinal tissues are significantly reduced with SIRT1 overexpression in these tissues (Fig.5E–H).

### SIRT1 overexpression prevents diabetes-induced micro-albuminuria and FN up-regulation in tissues

**Figure 6:**
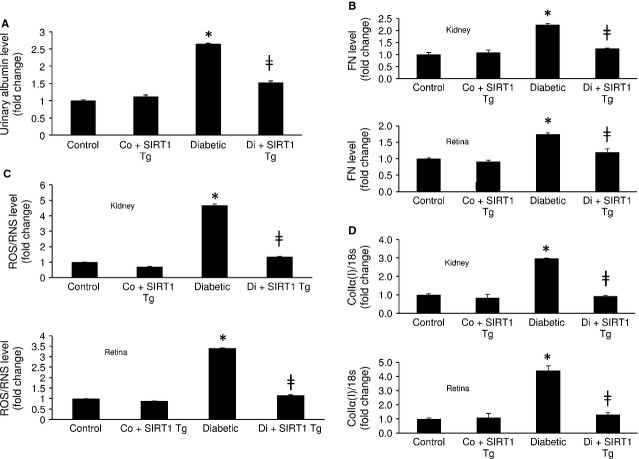
SIRT1 transgenic mice shows improved renal function and reduced oxidative stress and FN up-regulation with diabetes. (A) SIRT1 overexpression prevented diabetes-induced micro-albuminuria in mice. Furthermore, such overexpression averted diabetes-induced increased (B) collagen Iα(I) mRNA expression (C) FN protein and (D) total ROS/RNS levels in the renal and retinal tissues. Control = Non-diabetic wild-type, Co+SIRT1 Tg = SIRT1 transgenic mice non-diabetic, Diabetic = Wild-type diabetic, Di+SIRT1 Tg = SIRT1 transgenic mice diabetic, Co = Control, Di = Diabetic, * = significantly different from control, ╪ = significantly different from Diabetic. mRNA levels are expressed as a ratio of 18s. All data (mean ± SEM, *P* < 0.05) were normalized to Co; *n* = 8–10/group.

To examine the downstream consequence of SIRT1 mediated alteration of ET-1 and TGF-β1 in diabetes, we investigated urinary micro-albumin levels to assess renal function in these mice. Diabetic mice showed increased micro-albuminuria compared to controls indicating poor kidney function in these animals (Fig.6A). SIRT1 overexpression significantly reduced such micro-albuminuria in the transgenic diabetic animals (Fig.6A). Moreover histological analysis of renal tissues in these animals showed reduced mesangial expansion (a feature of diabetic nepthropathy) compared to the wild-type diabetic animals (Fig. S2C). Furthermore in the renal and retinal tissues of the diabetic animals such overexpression reduced diabetes-induced increased FN protein, total ROS/RNS and collagen Iα(I) mRNA expression levels (Fig.6B–D). These results suggest that SIRT1 has a protective role against diabetes-induced renal and retinal damages.

## Discussion

In this study, we have shown that SIRT1 regulates glucose-induced overexpression of ET-1 and TGF-β1 in the ECs. We have further shown that SIRT1 regulates ET-1 and TGF-β1 levels in the kidneys and retinas of diabetic animals. In addition, we have demonstrated such processes are regulated through transcriptional co-activator p300. Using adenoviral overexpression of SIRT1 and knockdown of SIRT1 with siRNA, we have directly demonstrated that glucose-induced ET-1 and TGF-β1 up-regulation can be markedly reduced by increasing the availability of SIRT1. In the kidneys and retinas of SIRT1 overexpressing transgenic mice we have further established the existence of such regulation in a STZ-induced type 1 model of diabetes. To be best of our knowledge, such SIRT1 mediated regulation of ET-1 and TGF-β1 in diabetic complications have not been shown earlier.

In diabetes, high oxidative stress due to hyperglycaemia causes DNA damage and activates several transcription and vasoactive factors altering crucial gene expressions 2,6,7. Here, we describe the role of SIRT1 causing protection against ET-1 and TGF-β1 induced endothelial damage in hyperglycaemia. We initially demonstrated SIRT1 down-regulation in parallel to ET-1 and TGF-β1 up-regulation in glucose exposed ECs. Then, we directly demonstrated its functional significance in terms of endothelial permeability and collagen Iα(I) expression and the relationship with p300 in these cells. In keeping with our earlier findings here we have shown that acting in opposing manner, p300 and SIRT1 regulate each other in hyperglycaemia. We further showed the functional significance of this mechanism in an animal model of diabetic microangiopathy.

Although there is no previous work showing SIRT1 mediated regulation of ET-1 or TGF-β1 expressions, SIRT1 has been found to regulate downstream effectors of TGF-β1 *via* deacetylation of SMAD7 29. This study demonstrates a SIRT1 mediated regulation of TGF-β1 expressions which signals through SMADs. One of the major glucose-induced endothelial dysfunctions includes augmented ECM protein production 2–14. Increased ECM proteins are deposited in the tissue which is manifested as structural changes such as basement membrane thickening, mesangial expansion *etc*. We and others have demonstrated glucose-induced increased collagen and FN synthesis in the endothelial cells and in retina, kidney and heart in diabetes 2–19. We have also demonstrated that glucose induced ECM protein up-regulation is mediated through p300-dependent histone acetylation and p300 binds to promoter regions of ET-1 and FN genes 15–19. In this study, we have shown one further step of p300 mediated regulation of ET-1 and TGF-β1 in ECs and tissues of diabetic animals. We have shown that by modulating SIRT1 we can prevent such glucose induced damages.

Endothelin 1 and TGF-β1 are two important cytokines playing key roles in tissue damage in diabetic complications. It is of interest to note that such SIRT1 induced regulation is mediated through p300. p300 as a transcriptional co-activator is potentially capable of altering multiple transcripts 15,18,19. This study shows that it is possible to target either SIRT1 or p300 to prevent diabetes-induced alterations. We have previously shown that p300 is regulated by miR-200b and it regulates miR-146a 24,26. Additionally, we have shown that miR-195 regulates SIRT1 expression 27. In this assumption we showed a ying-yang relationship between p300 and SIRT1. Hence a complex web connecting multiple such epigenetic processes involving acetylators, deacetylators, miRNAs and other undetermined epigenetic process may ultimately dictate expression of specific transcripts. It is also possible that SIRT1 may influence several other inflammatory cytokines which are of importance in the context of nephropathy and retinopathy and other chronic diabetic complications. The present study also does not exclude possible relationship of SIRT1 with other HATs or genes of anti-fibrotic/anti-oxidative properties. However, such roles of SIRT1 in these processes need to be established in future through specific experiments. Although the possibility of independent action of SIRT1 and p300 cannot be completely eliminated, our current study suggests that SIRT1 may act through p300 in the ECs to produce these transcripts. As ECs are an important target of glucose induced damage, the current study focused on to investigate the effect of hyperglycaemia on ECs. It is possible that similar alteration of SIRT1 may occur in other cell types, this was however beyond the scope of this article.

The pathogenic mechanisms leading to chronic diabetic complications are complex. Several vasoactive and growth factors are simultaneously activated in response to hyperglycaemia and an intricate interplay occurs among such factors 2–20,24–26. The findings of this study indicate that SIRT1 is a protective molecule in diabetic nephropathy and retinopathy. As loss of SIRT1 production represents an important event in the pathogenesis of chronic diabetic complications 20,27, SIRT1 holds the potential to be used as a drug target for treating such complications.

## Supporting Information

**Figure S1** Glucose induced increased (A) ET-1 and (B) TGF-β1 protein levels were normalized in the ECs with p300 siRNA transfection whereas p300 overexpression increased their level in NG.

**Figure S2** (A) Genotyping analyses of wild-type (WT) and SIRT1 transgenic mice (SIRT1 Tg) showed presence of a 200 bp band, thus confirming absence of Rd8 mutation in *Crb1* gene (detailed method at reference 28).
