# Supplementary figures and images for: SIRT1 reduction causes renal and retinal injury in diabetes through endothelin 1 and transforming growth factor β1
Source: J Cell Mol Med. 2015 Mar 6;19(8):1857–67. doi: 10.1111/jcmm.12557 (PMC4549036; doi:10.1111/jcmm.12557)

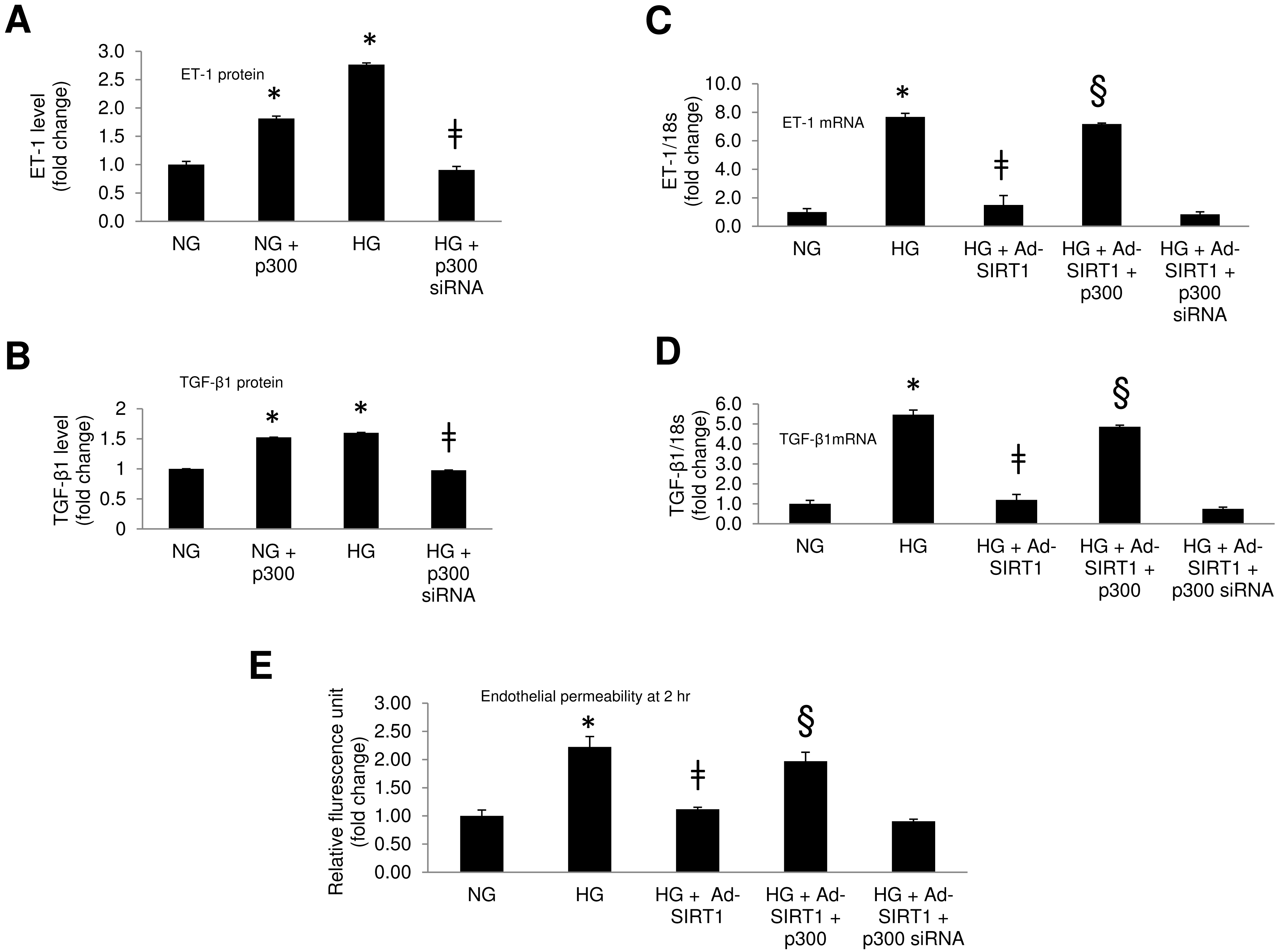

Supplement: Supplementary file 1 [file jcmm0019-1857-sd1.tif]

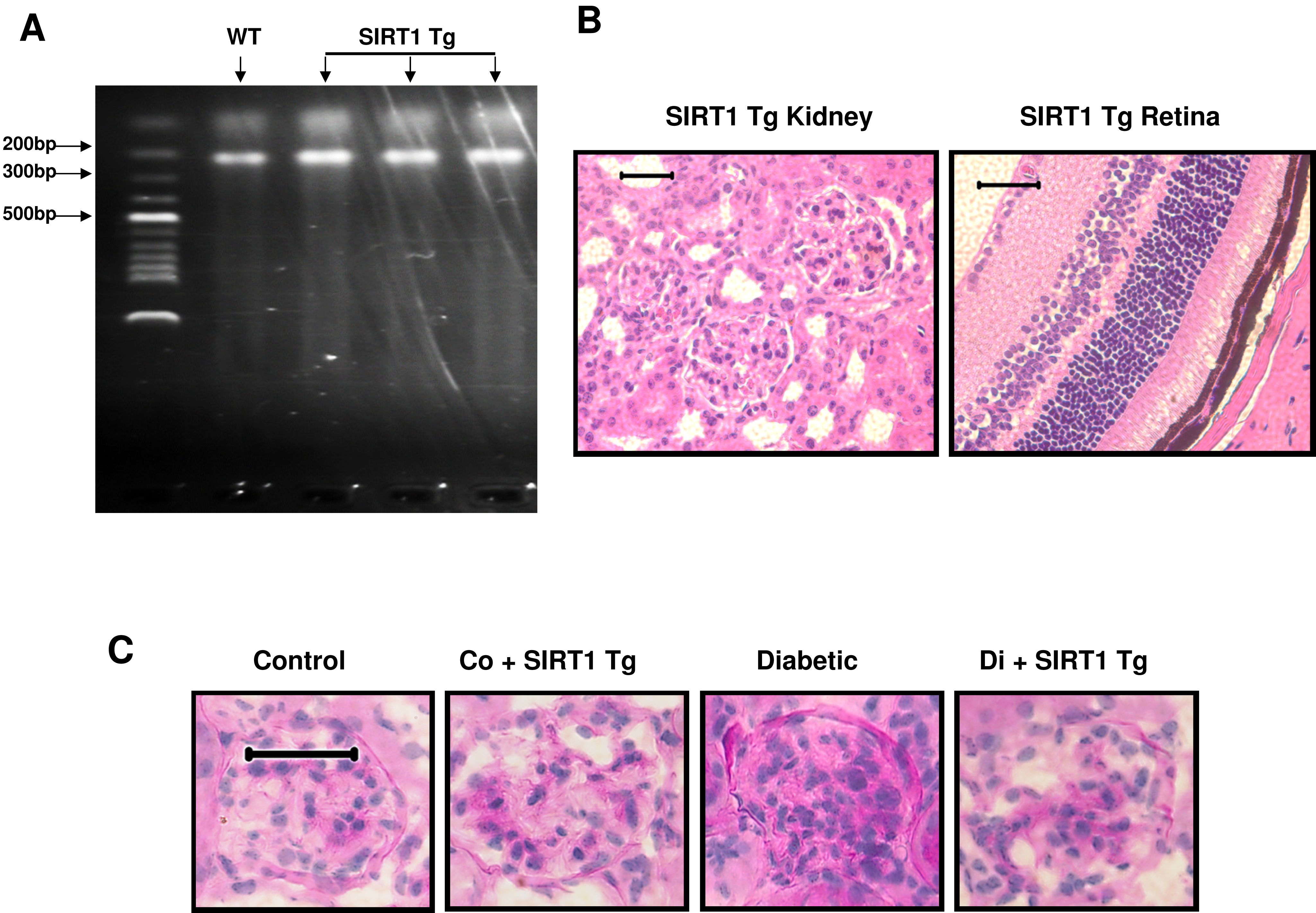

Supplement: Supplementary file 2 [file jcmm0019-1857-sd2.tif]
